# A case study on the relationship between risk assessment of scientific research projects and related factors under the Naive Bayesian algorithm

**DOI:** 10.1038/s41598-024-58341-y

**Published:** 2024-04-08

**Authors:** Xuying Dong, Wanlin Qiu

**Affiliations:** Institute of Policy Studies, Lingnan University, Tuen Mun 999077 Hong Kong, China

**Keywords:** Naive Bayesian algorithm, Scientific research projects, Risk assessment, Factor analysis, Probability estimation, Decision support, Data-driven decision-making, Mathematics and computing, Computer science

## Abstract

This paper delves into the nuanced dynamics influencing the outcomes of risk assessment (RA) in scientific research projects (SRPs), employing the Naive Bayes algorithm. The methodology involves the selection of diverse SRPs cases, gathering data encompassing project scale, budget investment, team experience, and other pertinent factors. The paper advances the application of the Naive Bayes algorithm by introducing enhancements, specifically integrating the Tree-augmented Naive Bayes (TANB) model. This augmentation serves to estimate risk probabilities for different research projects, shedding light on the intricate interplay and contributions of various factors to the RA process. The findings underscore the efficacy of the TANB algorithm, demonstrating commendable accuracy (average accuracy 89.2%) in RA for SRPs. Notably, budget investment (regression coefficient: 0.68, P < 0.05) and team experience (regression coefficient: 0.51, P < 0.05) emerge as significant determinants obviously influencing RA outcomes. Conversely, the impact of project size (regression coefficient: 0.31, P < 0.05) is relatively modest. This paper furnishes a concrete reference framework for project managers, facilitating informed decision-making in SRPs. By comprehensively analyzing the influence of various factors on RA, the paper not only contributes empirical insights to project decision-making but also elucidates the intricate relationships between different factors. The research advocates for heightened attention to budget investment and team experience when formulating risk management strategies. This strategic focus is posited to enhance the precision of RAs and the scientific foundation of decision-making processes.

## Introduction

Scientific research projects (SRPs) stand as pivotal drivers of technological advancement and societal progress in the contemporary landscape^1–3^. The dynamism of SRP success hinges on a multitude of internal and external factors^4^. Central to effective project management, Risk assessment (RA) in SRPs plays a critical role in identifying and quantifying potential risks. This process not only aids project managers in formulating strategic decision-making approaches but also enhances the overall success rate and benefits of projects. In a recent contribution, Salahuddin^5^ provides essential numerical techniques indispensable for conducting RAs in SRPs. Building on this foundation, Awais and Salahuddin^6^ delve into the assessment of risk factors within SRPs, notably introducing the consideration of activation energy through an exploration of the radioactive magnetohydrodynamic model. Further expanding the scope, Awais and Salahuddin^7^ undertake a study on the natural convection of coupled stress fluids. However, RA of SRPs confronts a myriad of challenges, underscoring the critical need for novel methodologies^8^. Primarily, the intricate nature of SRPs renders precise RA exceptionally complex and challenging. The project’s multifaceted dimensions, encompassing technology, resources, and personnel, are intricately interwoven, posing a formidable challenge for traditional assessment methods to comprehensively capture all potential risks^9^. Furthermore, the intricate and diverse interdependencies among various project factors contribute to the complexity of these relationships, thereby limiting the efficacy of conventional methods^10–12^. Traditional approaches often focus solely on the individual impact of diverse factors, overlooking the nuanced relationships that exist between them—an inherent limitation in the realm of RA for SRPs^13–15^.

The pursuit of a methodology capable of effectively assessing project risks while elucidating the intricate interplay of different factors has emerged as a focal point in SRPs management^16–18^. This approach necessitates a holistic consideration of multiple factors, their quantification in contributing to project risks, and the revelation of their correlations. Such an approach enables project managers to more precisely predict and respond to risks. Marx-Stoelting et al.^19^, current approaches for the assessment of environmental and human health risks due to exposure to chemical substances have served their purpose reasonably well. Additionally, Awais et al.^20^ highlights the significance of enthalpy changes in SRPs risk considerations, while Awais et al.^21^ delve into the comprehensive exploration of risk factors in Eyring-Powell fluid flow in magnetohydrodynamics, particularly addressing viscous dissipation and activation energy effects. The Naive Bayesian algorithm, recognized for its prowess in probability and statistics, has yielded substantial results in information retrieval and data mining in recent years^22^. Leveraging its advantages in classification and probability estimation, the algorithm presents a novel approach for RA of SRPs^23^. Integrating probability analysis into RA enables a more precise estimation of project risks by utilizing existing project data and harnessing the capabilities of the Naive Bayesian algorithms. This method facilitates a quantitative, statistical analysis of various factors, effectively navigating the intricate relationships between them, thereby enhancing the comprehensiveness and accuracy of RA for SRPs.

This paper seeks to employ the Naive Bayesian algorithm to estimate the probability of risks by carefully selecting distinct research project cases and analyzing multidimensional data, encompassing project scale, budget investment, and team experience. Concurrently, Multiple Linear Regression (MLR) analysis is applied to quantify the influence of these factors on the assessment results. The paper places particular emphasis on exploring the intricate interrelationships between different factors, aiming to provide a more specific and accurate reference framework for decision-making in SRPs management.

This paper introduces several innovations and contributions to the field of RA for SRPs:

- Comprehensive Consideration of Key Factors: Unlike traditional research that focuses on a single factor, this paper comprehensively considers multiple key factors, such as project size, budget investment, and team experience. This holistic analysis enhances the realism and thoroughness of RA for SRPs.
- Introduction of Tree-Enhanced Naive Bayes Model: The naive Bayes algorithm is introduced and further improved through the proposal of a tree-enhanced naive Bayes model. This algorithm exhibits unique advantages in handling uncertainty and complexity, thereby enhancing its applicability and accuracy in the RA of scientific and technological projects.
- Empirical Validation: The effectiveness of the proposed method is not only discussed theoretically but also validated through empirical cases. The analysis of actual cases provides practical support and verification, enhancing the credibility of the research results.
- Application of MLR Analysis: The paper employs MLR analysis to delve into the impact of various factors on RA. This quantitative analysis method adds specificity and operability to the research, offering a practical decision-making basis for scientific and technological project management.
- Discovery of New Connections and Interactions: The paper uncovers novel connections and interactions, such as the compensatory role of team experience for budget-related risks and the impact of the interaction between project size and budget investment on RA results. These insights provide new perspectives for decision-making in technology projects, contributing significantly to the field of RA for SRPs in terms of both importance and practical value.

The paper is structured as follows: “Introduction” briefly outlines the significance of RA for SRPs. Existing challenges within current research are addressed, and the paper’s core objectives are elucidated. A distinct emphasis is placed on the innovative aspects of this research compared to similar studies. The organizational structure of the paper is succinctly introduced, providing a brief overview of each section’s content. “Literature review” provides a comprehensive review of relevant theories and methodologies in the domain of RA for SRPs. The current research landscape is systematically examined, highlighting the existing status and potential gaps. Shortcomings in previous research are analyzed, laying the groundwork for the paper’s motivation and unique contributions. “Research methodology” delves into the detailed methodologies employed in the paper, encompassing data collection, screening criteria, preprocessing steps, and more. The tree-enhanced naive Bayes model is introduced, elucidating specific steps and the purpose behind MLR analysis. “Results and discussion” unfolds the results and discussions based on selected empirical cases. The representativeness and diversity of these cases are expounded upon. An in-depth analysis of each factor’s impact and interaction in the context of RA is presented, offering valuable insights. “Discussion” succinctly summarizes the entire research endeavor. Potential directions for further research and suggestions for improvement are proposed, providing a thoughtful conclusion to the paper.

## Literature review

### A review of RA for SRPs

In recent years, the advancement of SRPs management has led to the evolution of various RA methods tailored for SRPs. The escalating complexity of these projects poses a challenge for traditional methods, often falling short in comprehensively considering the intricate interplay among multiple factors and yielding incomplete assessment outcomes. Scholars, recognizing the pivotal role of factors such as project scale, budget investment, and team experience in influencing project risks, have endeavored to explore these dynamics from diverse perspectives. Siyal et al.^24^ pioneered the development and testing of a model geared towards detecting SRPs risks. Chen et al.^25^ underscored the significance of visual management in SRPs risk management, emphasizing its importance in understanding and mitigating project risks. Zhao et al.^26^ introduced a classic approach based on cumulative prospect theory, offering an optional method to elucidate researchers’ psychological behaviors. Their study demonstrated the enhanced rationality achieved by utilizing the entropy weight method to derive attribute weight information under Pythagorean fuzzy sets. This approach was then applied to RA for SRPs, showcasing a model grounded in the proposed methodology. Suresh and Dillibabu^27^ proposed an innovative hybrid fuzzy-based machine learning mechanism tailored for RA in software projects. This hybrid scheme facilitated the identification and ranking of major software project risks, thereby supporting decision-making throughout the software project lifecycle. Akhavan et al.^28^ introduced a Bayesian network modeling framework adept at capturing project risks by calculating the uncertainty of project net present value. This model provided an effective means for analyzing risk scenarios and their impact on project success, particularly applicable in evaluating risks for innovative projects that had undergone feasibility studies.

### A review of factors affecting SRPs

Within the realm of SRPs management, the assessment and proficient management of project risks stand as imperative components. Consequently, a range of studies has been conducted to explore diverse methods and models aimed at enhancing the comprehension and decision support associated with project risks. Guan et al.^29^ introduced a new risk interdependence network model based on Monte Carlo simulation to support decision-makers in more effectively assessing project risks and planning risk management actions. They integrated interpretive structural modeling methods into the model to develop a hierarchical project risk interdependence network based on identified risks and their causal relationships. Vujović et al.^30^ provided a new method for research in project management through careful analysis of risk management in SRPs. To confirm the hypothesis, the study focused on educational organizations and outlined specific project management solutions in business systems, thereby improving the business and achieving positive business outcomes. Muñoz-La Rivera et al.^31^ described and classified the 100 identified factors based on the dimensions and aspects of the project, assessed their impact, and determined whether they were shaping or directly affecting the occurrence of research project accidents. These factors and their descriptions and classifications made significant contributions to improving the security creation of the system and generating training and awareness materials, fostering the development of a robust security culture within organizations. Nguyen et al. concentrated on the pivotal risk factors inherent in design-build projects within the construction industry. Effective identification and management of these factors enhanced project success and foster confidence among owners and contractors in adopting the design-build approach^32^. Their study offers valuable insights into RA in project management and the adoption of new contract forms. Nguyen and Le delineated risk factors influencing the quality of 20 civil engineering projects during the construction phase^33^. The top five risks identified encompass poor raw material quality, insufficient worker skills, deficient design documents and drawings, geographical challenges at construction sites, and inadequate capabilities of main contractors and subcontractors. Meanwhile, Nguyen and Phu Pham concentrated on office building projects in Ho Chi Minh City, Vietnam, to pinpoint key risk factors during the construction phase^34^. These factors were classified into five groups based on their likelihood and impact: financial, management, schedule, construction, and environmental. Findings revealed that critical factors affecting office building projects encompassed both natural elements (e.g., prolonged rainfall, storms, and climate impacts) and human factors (e.g., unstable soil, safety behavior, owner-initiated design changes), with schedule-related risks exerting the most significant influence during the construction phase of Ho Chi Minh City’s office building projects. This provides construction and project management practitioners with fresh insights into risk management, aiding in the comprehensive identification, mitigation, and management of risk factors in office building projects.

### Summary

While existing research has made notable strides in RA for SRPs, certain limitations persist. These studies limitations in quantifying the degree of influence of various factors and analyzing their interrelationships, thereby falling short of offering specific and actionable recommendations. Traditional methods, due to their inherent limitations, struggle to precisely quantify risk degrees and often overlook the intricate interplay among multiple factors. Consequently, there is an urgent need for a comprehensive method capable of quantifying the impact of diverse factors and revealing their correlations. In response to this exigency, this paper introduces the TANB model. The unique advantages of this algorithm in the RA of scientific and technological projects have been fully realized. Tailored to address the characteristics of uncertainty and complexity, the model represents a significant leap forward in enhancing applicability and accuracy. In comparison with traditional methods, the TANB model exhibits greater flexibility and a heightened ability to capture dependencies between features, thereby elevating the overall performance of RA. This innovative method emerges as a more potent and reliable tool in the realm of scientific and technological project management, furnishing decision-makers with more comprehensive and accurate support for RA.

## Research methodology

This paper centers on the latest iteration of ISO 31000, delving into the project risk management process and scrutinizing the RA for SRPs and their intricate interplay with associated factors. ISO 31000, an international risk management standard, endeavors to furnish businesses, organizations, and individuals with a standardized set of risk management principles and guidelines, defining best practices and establishing a common framework. The paper unfolds in distinct phases aligned with ISO 31000:

- Risk Identification: Employing data collection and preparation, a spectrum of factors related to project size, budget investment, team member experience, project duration, and technical difficulty were identified.
- RA: Utilizing the Naive Bayes algorithm, the paper conducts RA for SRPs, estimating the probability distribution of various factors influencing RA results.
- Risk Response: The application of the Naive Bayes model is positioned as a means to respond to risks, facilitating the formulation of apt risk response strategies based on calculated probabilities.
- Monitoring and Control: Through meticulous data collection, model training, and verification, the paper illustrates the steps involved in monitoring and controlling both data and models. Regular monitoring of identified risks and responses allows for adjustments when necessary.
- Communication and Reporting: Maintaining effective communication throughout the project lifecycle ensures that stakeholders comprehend the status of project risks. Transparent reporting on discussions and outcomes contributes to an informed project environment.

### Data collection and preparation

In this paper, a meticulous approach is undertaken to select representative research project cases, adhering to stringent screening criteria. Additionally, a thorough review of existing literature is conducted and tailored to the practical requirements of SRPs management. According to Nguyen et al., these factors play a pivotal role in influencing the RA outcomes of SRPs^35^. Furthermore, research by He et al. underscored the significant impact of team members’ experience on project success^36^. Therefore, in alignment with our research objectives and supported by the literature, this paper identifies variables such as project scale, budget investment, team member experience, project duration, and technical difficulty as the focal themes. To ensure the universality and scientific rigor of our findings, the paper adheres to stringent selection criteria during the project case selection process. After preliminary screening of SRPs completed in the past 5 years, considering factors such as project diversity, implementation scales, and achieved outcomes, five representative projects spanning diverse fields, including engineering, medicine, and information technology, are ultimately selected. These project cases are chosen based on their capacity to represent various scales and types of SRPs, each possessing a typical risk management process, thereby offering robust and comprehensive data support for our study. The subsequent phase involves detailed data collection on each chosen project, encompassing diverse dimensions such as project scale, budget investment, team member experience, project cycle, and technical difficulty. The collected data undergo meticulous preprocessing to ensure data quality and reliability. The preprocessing steps comprised data cleaning, addressing missing values, handling outliers, culminating in the creation of a self-constructed dataset. The dataset encompasses over 500 SRPs across diverse disciplines and fields, ensuring statistically significant and universal outcomes. Particular emphasis is placed on ensuring dataset diversity, incorporating projects of varying scales, budgets, and team experience levels. This comprehensive coverage ensures the representativeness and credibility of the study on RA in SRPs. New influencing factors are introduced to expand the research scope, including project management quality (such as time management and communication efficiency), historical success rate, industry dynamics, and market demand. Detailed definitions and quantifications are provided for each new variable to facilitate comprehensive data processing and analysis. For project management quality, consideration is given to time management accuracy and communication frequency and quality among team members. Historical success rate is determined by reviewing past project records and outcomes. Industry dynamics are assessed by consulting the latest scientific literature and patent information. Market demand is gauged through market research and user demand surveys. The introduction of these variables enriches the understanding of RA in SRPs and opens up avenues for further research exploration.

At the same time, the collected data are integrated and coded in order to apply Naive Bayes algorithm and MLR analysis. For cases involving qualitative data, this paper uses appropriate coding methods to convert it into quantitative data for processing in the model. For example, for the qualitative feature of team member experience, numerical values are used to represent different experience levels, such as 0 representing beginners, 0 representing intermediate, and 2 representing advanced. The following is a specific sample data set example (Table 1). It shows the processed structured data, and the values in the table represent the specific characteristics of each project.

**Table 1:** Example of dataset encoding.

Project name	Field	Project scale	Budget investment	Team member experience	Project cycle	Technical difficulty
Project A	Engineering	100	500	2	12	3
Project B	Medicine	50	300	1	8	2
Project C	Information technology	200	800	2	18	4
Project D	Engineering	150	600	3	15	3
Project E	Medicine	80	450	2	10	2

### Establishment of naive Bayesian model

**Figure 1:**
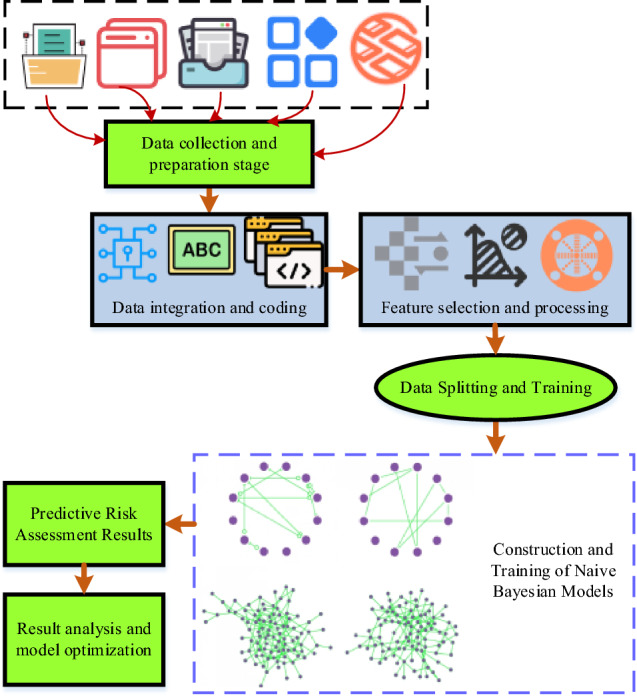
Naive Bayesian algorithm process.

The Naive Bayesian algorithm, a probabilistic and statistical classification method renowned for its effectiveness in analyzing and predicting multi-dimensional data, is employed in this paper to conduct the RA for SRPs. The application of the Naive Bayesian algorithm to RA for SRPs aims to discern the influence of various factors on the outcomes of RA. The Naive Bayesian algorithm, depicted in Fig. 1, operates on the principles of Bayesian theorem, utilizing posterior probability calculations for classification tasks. The fundamental concept of this algorithm hinges on the assumption of independence among different features, embodying the “naivety” hypothesis. In the context of RA for SRPs, the Naive Bayesian algorithm is instrumental in estimating the probability distribution of diverse factors affecting the RA results, thereby enhancing the precision of risk estimates. In the Naive Bayesian model, the initial step involves the computation of posterior probabilities for each factor, considering the given RA result conditions. Subsequently, the category with the highest posterior probability is selected as the predictive outcome.

In Fig. 1, the data collection process encompasses vital project details such as project scale, budget investment, team member experience, project cycle, technical difficulty, and RA results. This meticulous collection ensures the integrity and precision of the dataset. Subsequently, the gathered data undergoes integration and encoding to convert qualitative data into quantitative form, facilitating model processing and analysis. Tailored to specific requirements, relevant features are chosen for model construction, accompanied by essential preprocessing steps like standardization and normalization. The dataset is then partitioned into training and testing sets, with the model trained on the former and its performance verified on the latter. Leveraging the training data, a Naive Bayesian model is developed to estimate probability distribution parameters for various features across distinct categories. Ultimately, the trained model is employed to predict new project features, yielding RA results.

Naive Bayesian models, in this context, are deployed to forecast diverse project risk levels. Let X symbolize the feature vector, encompassing project scale, budget investment, team member experience, project cycle, and technical difficulty. The objective is to predict the project’s risk level, denoted as Y. Y assumes discrete values representing distinct risk levels. Applying the Bayesian theorem, the posterior probability P(Y|X) is computed, signifying the probability distribution of projects falling into different risk levels given the feature vector X. The fundamental equation governing the Naive Bayesian model is expressed as:

1 $$P(Y|X)=\frac{P(X|Y)\cdot P(Y)}{P(X)}$$

In Eq. (1), P(Y|X) represents the posterior probability, denoting the likelihood of the project belonging to a specific risk level. P(X|Y) signifies the class conditional probability, portraying the likelihood of the feature vector X occurring under known risk level conditions. P(Y) is the prior probability, reflecting the antecedent likelihood of the project pertaining to a particular risk level. P(X) acts as the evidence factor, encapsulating the likelihood of the feature vector X occurring.

The Naive Bayes, serving as the most elementary Bayesian network classifier, operates under the assumption of attribute independence given the class label *c*, as expressed in Eq. (2):

2 $$P\left( {x|c} \right) = \prod\limits_{i = 1}^{n} {P\left( {x_{i} |c} \right)}$$

The classification decision formula for Naive Bayes is articulated in Eq. (3):

3 $$c*_{NB} = \arg \mathop {\max }\limits_{{c \in \Omega_{C} }} P\left( c \right)\prod\limits_{i = 1}^{n} {P\left( {x_{i} |c} \right)}$$

The Naive Bayes model, rooted in the assumption of conditional independence among attributes, often encounters deviations from reality. To address this limitation, the Tree-Augmented Naive Bayes (TANB) model extends the independence assumption by incorporating a first-order dependency maximum-weight spanning tree. TANB introduces a tree structure that more comprehensively models relationships between features, easing the constraints of the independence assumption and concurrently mitigating issues associated with multicollinearity. This extension bolsters its efficacy in handling intricate real-world data scenarios. TANB employs conditional mutual information $I(X_{i} ;X_{j} |C)$ to gauge the dependency between attributes $X_{j}$ and $X_{i}$, thereby constructing the maximum weighted spanning tree. In TANB, any attribute variable $X_{i}$ is permitted to have at most one other attribute variable as its parent node, expressed as $Pa\left( {X_{i} } \right) \le 2$. The joint probability $P_{con} \left( {x,c} \right)$ undergoes transformation using Eq. (4):

4 $$P_{con} \left( {x,c} \right) = P\left( c \right)P\left( {x_{r} |c} \right)\prod\limits_{i = 1}^{n} {P\left( {x_{i} |c,x_{j\left( i \right)} } \right)}$$

In Eq. (4), $x_{r}$ refers to the root node, which can be expressed as Eq. (5):

5 $$x_{j\left( i \right)} = Pa\left( {X_{i} } \right)/C,\quad i \ne r$$

TANB classification decision equation is presented below:

6 $$c*_{TAN} = \arg \mathop {\max }\limits_{{c \in \Omega_{C} }} P\left( c \right)P\left( {x_{r} |c} \right)\prod\limits_{i = 1}^{n} {P\left( {x_{i} |c,x_{j\left( i \right)} } \right)}$$

In the RA of SRPs, normal distribution parameters, such as mean (μ) and standard deviation (σ), are estimated for each characteristic dimension (project scale, budget investment, team member experience, project cycle, and technical difficulty). This estimation allows the calculation of posterior probabilities for projects belonging to different risk levels under given feature vector conditions. For each feature dimension ${X}_{i}$, the mean ${mu}_{i,j}$ and standard deviation ${{\text{sigma}}}_{i,j}$ under each risk level are computed, where *i* represents the feature dimension, and *j* denotes the risk level. Parameter estimation employs the maximum likelihood method, and the specific calculations are as follows.

7 $${\mu }_{i,j}=\frac{1}{{N}_{j}}{\sum }_{k=1}^{{N}_{j}}{x}_{i,k}$$

8 $${\sigma }_{i,j}=\sqrt{\frac{1}{{N}_{j}}{\sum }_{k=1}^{{N}_{j}}({x}_{i,k}-{\mu }_{i,j}{)}^{2}}$$

In Eqs. (7) and (8), ${N}_{j}$ represents the number of projects belonging to risk level *j*. ${x}_{i,k}$ denotes the value of the *k*-th item in the feature dimension *i*. Finally, under a given feature vector, the posterior probability of a project with risk level *j* is calculated as Eq. (9).

9 $$P(Y=j\mid X)=\frac{1}{Z}\cdot P(Y=j)\cdot {\prod }_{i=1}^{d}P({X}_{i}\mid Y=j)$$

In Eq. (9), *d* represents the number of feature dimensions, and *Z* is the normalization factor. $P(Y=j)$ represents the prior probability of category *j*. $P({X}_{i}\mid Y=j)$ represents the normal distribution probability density function of feature dimension *i* under category *j*. The risk level of a project can be predicted by calculating the posterior probabilities of different risk levels to achieve RA for SRPs.

This paper integrates the probability estimation of the Naive Bayes model with actual project risk response strategies, enabling a more flexible and targeted response to various risk scenarios. Such integration offers decision support to project managers, enhancing their ability to address potential challenges effectively and ultimately improving the overall success rate of the project. This underscores the notion that risk management is not solely about problem prevention but stands as a pivotal factor contributing to project success.

### MLR analysis

MLR analysis is used to validate the hypothesis to deeply explore the impact of various factors on RA of SRPs. Based on the previous research status, the following research hypotheses are proposed.

Hypothesis 1: There is a positive relationship among project scale, budget investment, and team member experience and RA results. As the project scale, budget investment, and team member experience increase, the RA results also increase.

Hypothesis 2: There is a negative relationship between the project cycle and the RA results. Projects with shorter cycles may have higher RA results.

Hypothesis 3: There is a complex relationship between technical difficulty and RA results, which may be positive, negative, or bidirectional in some cases. Based on these hypotheses, an MLR model is established to analyze the impact of factors, such as project scale, budget investment, team member experience, project cycle, and technical difficulty, on RA results. The form of an MLR model is as follows.

10 $$Y={\beta }_{0}+{\beta }_{1}{X}_{1}+{\beta }_{2}{X}_{2}+{\beta }_{3}{X}_{3}+{\beta }_{4}{X}_{4}+{\beta }_{5}{X}_{5}+\epsilon$$

**Figure 2:**
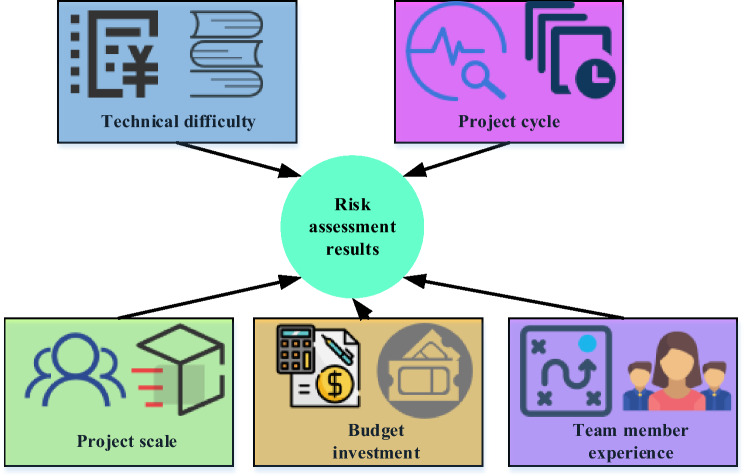
Schematic diagram of an MLR model.

In Eq. (10), *Y* represents the RA result (dependent variable). ${X}_{1}$ to ${X}_{5}$ represent factors, such as project scale, budget investment, team member experience, project cycle, and technical difficulty (independent variables). ${\beta }_{0}$ to ${\beta }_{5}$ are the regression coefficients, which represent the impact of various factors on the RA results. $\epsilon$ represents a random error term. The model structure is shown in Fig. 2.

In Fig. 2, the MLR model is employed to scrutinize the influence of various independent variables on the outcomes of RA. In this specific context, the independent variables encompass project size, budget investment, team member experience, project cycle, and technical difficulty, all presumed to impact the project’s RA results. Each independent variable is denoted as a node in the model, with arrows depicting the relationships between these factors. In an MLR model, the arrow direction signifies causality, illustrating the influence of an independent variable on the dependent variable.

When conducting MLR analysis, it is necessary to estimate the parameter $\upbeta$ in the regression model. These parameters determine the relationship between the independent and dependent variables. Here, the Ordinary Least Squares (OLS) method is applied to estimate these parameters. The OLS method is a commonly used parameter estimation method aimed at finding parameter values that minimize the sum of squared residuals between model predictions and actual observations. The steps are as follows. Firstly, based on the general form of an MLR model, it is assumed that there is a linear relationship between the independent and dependent variables. It can be represented by a linear equation, which includes regression coefficients β and the independent variable X. For each observation value, the difference between its predicted and actual values is calculated, which is called the residual. Residual ${e}_{i}$ can be expressed as:

11 $${e}_{i}={Y}_{i}-{\widehat{Y}}_{i}$$

In Eq. (11), ${Y}_{i}$ is the actual observation value, and ${\widehat{Y}}_{i}$ is the value predicted by the model. The goal of the OLS method is to adjust the regression coefficients $\upbeta$ to minimize the sum of squared residuals of all observations. This can be achieved by solving an optimization problem, and the objective function is the sum of squared residuals.

12 $${\text{minimize}}{\sum }_{i=1}^{n}{e}_{i}^{2}=={\sum }_{i=1}^{n}({Y}_{i}-\widehat{{Y}_{i}}{)}^{2}$$

Then, the estimated value of the regression coefficient $\upbeta$ that minimizes the sum of squared residuals can be obtained by taking the derivative of the objective function and making the derivative zero. The estimated values of the parameters can be obtained by solving this system of equations. The final estimated regression coefficient can be expressed as:

13 $$\widehat{\beta }=({X}^{T}X{)}^{-1}{X}^{T}Y$$

In Eq. (13), *X* represents the independent variable matrix. *Y* represents the dependent variable vector. $({X}^{T}X{)}^{-1}$ is the inverse of a matrix, and $\widehat{\beta }$ is a parameter estimation vector.

Specifically, solving for the estimated value of regression coefficient $\upbeta$ requires matrix operation and statistical analysis. Based on the collected project data, substitute it into the model and calculate the residual. Then, the steps of the OLS method are used to obtain parameter estimates. These parameter estimates are used to establish an MLR model to predict RA results and further analyze the influence of different factors.

The degree of influence of different factors on the RA results can be determined by analyzing the value of the regression coefficient β. A positive $\upbeta$ value indicates that the factor has a positive impact on the RA results, while a negative $\upbeta$ value indicates that the factor has a negative impact on the RA results. Additionally, hypothesis testing can determine whether each factor is significant in the RA results.

The TANB model proposed in this paper extends the traditional naive Bayes model by incorporating conditional dependencies between attributes to enhance the representation of feature interactions. While the traditional naive Bayes model assumes feature independence, real-world scenarios often involve interdependencies among features. To address this, the TANB model is introduced. The TANB model introduces a tree structure atop the naive Bayes model to more accurately model feature relationships, overcoming the limitation of assuming feature independence. Specifically, the TANB model constructs a maximum weight spanning tree to uncover conditional dependencies between features, thereby enabling the model to better capture feature interactions.

### Assessment indicators

To comprehensively assess the efficacy of the proposed TANB model in the RA for SRPs, a self-constructed dataset serves as the data source for this experimental evaluation, as outlined in Table 1. The dataset is segregated into training (80%) and test sets (20%). These indicators cover the accuracy, precision, recall rate, F1 value, and Area Under the Curve (AUC) Receiver Operating Characteristic (ROC) of the model. The following are the definitions and equations for each assessment indicator. Accuracy is the proportion of correctly predicted samples to the total number of samples. Precision is the proportion of Predicted Positive (PP) samples to actual positive samples. The recall rate is the proportion of correctly PP samples among the actual positive samples. The F1 value is the harmonic average of precision and recall, considering the precision and comprehensiveness of the model. The area under the ROC curve measures the classification performance of the model, and a larger AUC value indicates better model performance. The ROC curve suggests the relationship between True Positive Rate and False Positive Rate under different thresholds. The AUC value can be obtained by accumulating the area of each small rectangle under the ROC curve. The confusion matrix is used to display the prediction property of the model in different categories, including True Positive (TP), True Negative (TN), False Positive (FP), and False Negative (FN).

14 $$Accuracy=\frac{TP+TN}{TP+TN+FP+FN}$$

15 $$Precision=\frac{TP}{TP+FP}$$

16 $$Recall=\frac{TP}{TP+FN}$$

17 $$F1=\frac{2*Precision*Recall}{Precision+Recall}$$

The performance of TANB in RA for SRPs can be comprehensively assessed to understand the advantages, disadvantages, and applicability of the model more comprehensively by calculating the above assessment indicators.

## Results and discussion

### Accuracy analysis of Naive Bayesian algorithm

**Figure 3:**
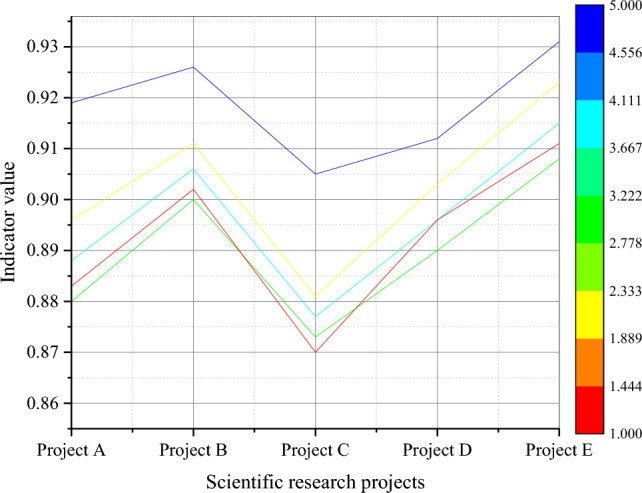
Performance assessment of TANB algorithm on different projects.

On the dataset of this paper, Fig. 3 reveals the performance of TANB algorithm under different assessment indicators.

**Figure 4:**
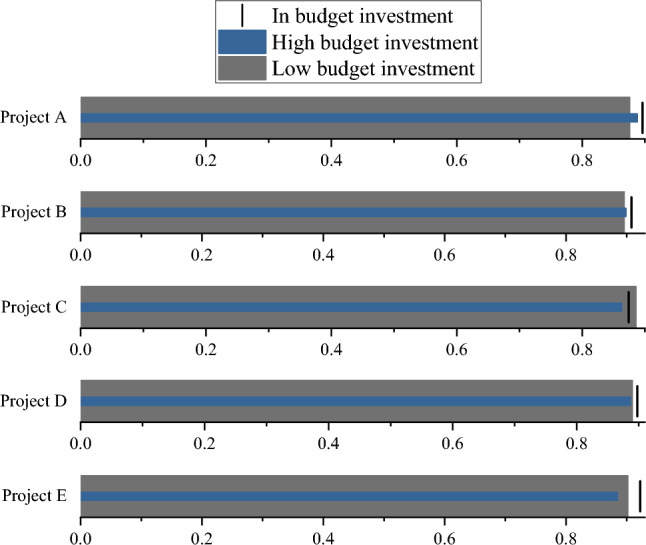
Prediction accuracy of TANB algorithm on different budget investments.

**Figure 5:**
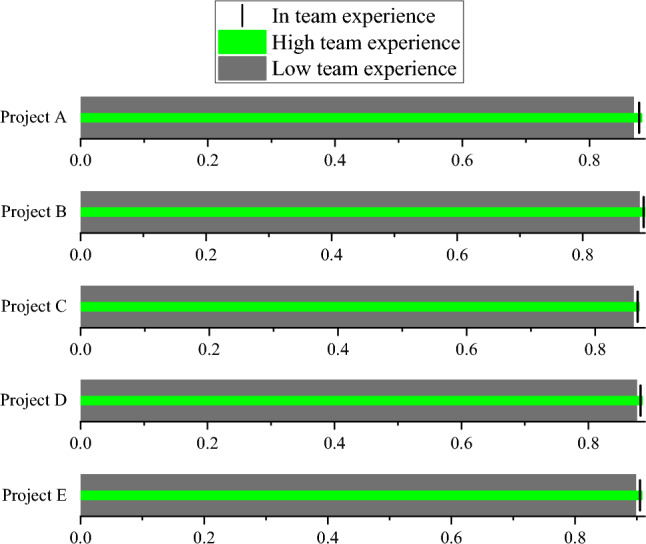
Prediction accuracy of TANB algorithm on different team experiences.

**Figure 6:**
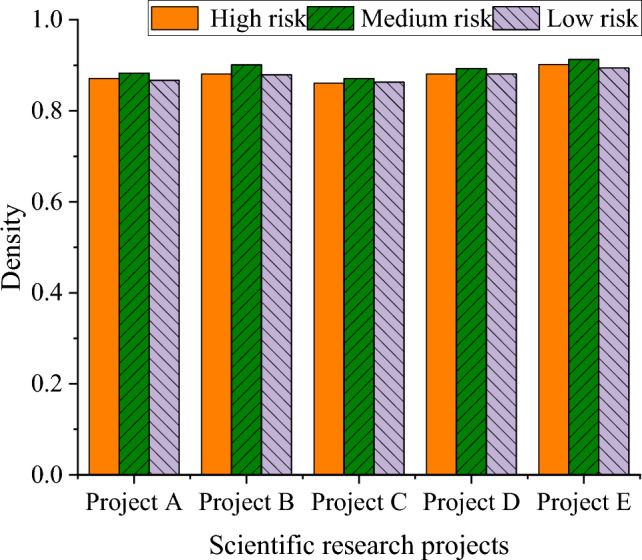
Prediction accuracy of TANB algorithm at different risk levels.

**Figure 7:**
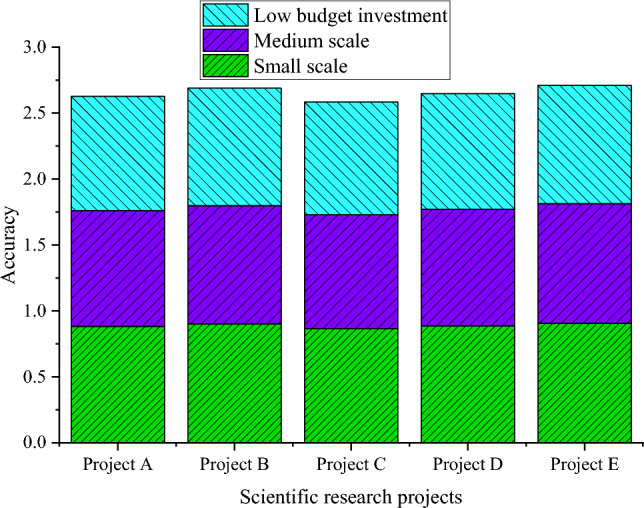
Prediction accuracy of TANB algorithm on different project scales.

From Fig. 3, the TANB algorithm performs well in various projects, ranging from 0.87 to 0.911 in accuracy. This means that the overall accuracy of the model in predicting project risks is quite high. The precision also maintains a high level in various projects, ranging from 0.881 to 0.923, indicating that the model performs well in classifying high-risk categories. The recall rate ranges from 0.872 to 0.908, indicating that the model can effectively capture high-risk samples. Meanwhile, the AUC values in each project are relatively high, ranging from 0.905 to 0.931, which once again emphasizes the effectiveness of the model in risk prediction. From multiple assessment indicators, such as accuracy, precision, recall, F1 value, and AUC, the TANB algorithm has shown good risk prediction performance in representative projects. The performance assessment results of the TANB algorithm under different feature dimensions are plotted in Figs. 4, 5, 6 and 7.

From Figs. 4, 5, 6 and 7, as the level of budget investment increases, the accuracy of most projects also shows an increasing trend. Especially in cases of high budget investment, the accuracy of the project is generally high. This may mean that a higher budget investment helps to reduce project risks, thereby improving the prediction accuracy of the TANB algorithm. It can be observed that team experience also affects the accuracy of the model. Projects with high team experience exhibit higher accuracy in TANB algorithms. This may indicate that experienced teams can better cope with project risks to improve the performance of the model. When budget investment and team experience are low, accuracy is relatively low. This may imply that budget investment and team experience can complement each other to affect the model performance.

There are certain differences in the accuracy of projects under different risk levels. Generally speaking, the accuracy of high-risk and medium-risk projects is relatively high, while the accuracy of low-risk projects is relatively low. This may be because high-risk and medium-risk projects require more accurate predictions, resulting in higher accuracy. Similarly, project scale also affects the performance of the model. Large-scale and medium-scale projects exhibit high accuracy in TANB algorithms, while small-scale projects have relatively low accuracy. This may be because the risks of large-scale and medium-scale projects are easier to identify and predict to promote the performance of the model. In high-risk and large-scale projects, accuracy is relatively high. This may indicate that the impact of project scale is more significant in specific risk scenarios.

**Figure 8:**
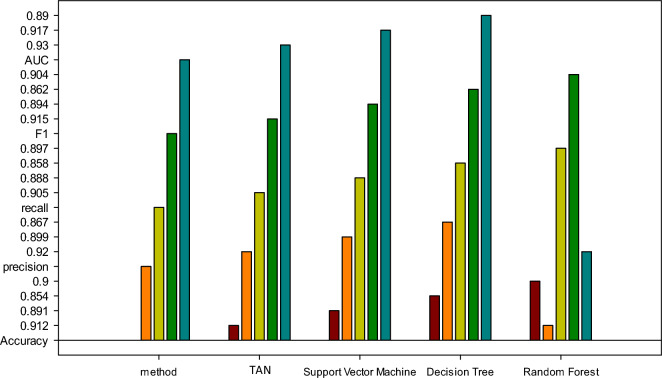
Performance comparison of different algorithms in RA of SRPs.

Figure 8 further compares the performance of the TANB algorithm proposed here with other similar algorithms.

As depicted in Fig. 8, the TANB algorithm attains an accuracy and precision of 0.912 and 0.920, respectively, surpassing other algorithms. It excels in recall rate and F1 value, registering 0.905 and 0.915, respectively, outperforming alternative algorithms. These findings underscore the proficiency of the TANB algorithm in comprehensively identifying high-risk projects while sustaining high classification accuracy. Moreover, the algorithm achieves an AUC of 0.930, indicative of its exceptional predictive prowess in sample classification. Thus, the TANB algorithm exhibits notable potential for application, particularly in scenarios demanding the recognition and comprehensiveness requisite for high-risk project identification. The evaluation results of the TANB model in predicting project risk levels are presented in Table 2:

**Table 2:** Evaluation results of the TANB model in predicting project risk level indicators.

Model	Accuracy	Precision	Recall
Naive Bayes	0.87	0.881	0.872
TANB	0.911	0.923	0.908

Table 2 demonstrates that the TANB model surpasses the traditional Naive Bayes model across multiple evaluation metrics, including accuracy, precision, and recall. This signifies that, by accounting for feature interdependence, the TANB model can more precisely forecast project risk levels. Furthermore, leveraging the model’s predictive outcomes, project managers can devise tailored risk mitigation strategies corresponding to various risk scenarios. For example, in high-risk projects, more assertive measures can be implemented to address risks, while in low-risk projects, risks can be managed more cautiously. This targeted risk management approach contributes to enhancing project success rates, thereby ensuring the seamless advancement of SRPs.

The exceptional performance of the TANB model in specific scenarios derives from its distinctive characteristics and capabilities. Firstly, compared to traditional Naive Bayes models, the TANB model can better capture the dependencies between attributes. In project RA, project features often exhibit complex interactions. The TANB model introduces first-order dependencies between attributes, allowing features to influence each other, thereby more accurately reflecting real-world situations and improving risk prediction precision. Secondly, the TANB model demonstrates strong adaptability and generalization ability in handling multidimensional data. SRPs typically involve data from multiple dimensions, such as project scale, budget investment, and team experience. The TANB model effectively processes these multidimensional data, extracts key information, and achieves accurate RA for projects. Furthermore, the paper explores the potential of using hybrid models or ensemble learning methods to further enhance model performance. By combining other machine learning algorithms, such as random forests and support vector regressors with sigmoid kernel, through ensemble learning, the shortcomings of individual models in specific scenarios can be overcome, thus improving the accuracy and robustness of RA. For example, in the study, we compared the performance of the TANB model with other algorithms in RA, as shown in Table 3.

**Table 3:** Performance of the TANB model and other algorithms in RA.

Model	Accuracy	Precision	Recall	F1 value	AUC value
TANB Model	0.912	0.920	0.905	0.915	0.930
Random Forest Model	0.901	0.915	0.895	0.905	0.920
Support Vector Regressor with Sigmoid Kernel	0.895	0.910	0.890	0.900	0.915

Table 3 illustrates that the TANB model surpasses other models in terms of accuracy, precision, recall, F1 value, and AUC value, further confirming its superiority and practicality in RA. Therefore, the TANB model holds significant application potential in SRPs, offering effective decision support for project managers to better evaluate and manage project risks, thereby enhancing the likelihood of project success.

### Analysis of the degree of influence of different factors

Table 4 analyzes the degree of influence and interaction of different factors.

**Table 4:** Regression analysis of the degree of influence and interaction of different factors.

Factor	Degree of influence (regression coefficient)	Standard error	t value	P value (P < 0.05)	Interaction and the influence of other factors
Budget investment	0.68	0.042	16.18	< 0.001	Team experience (0.51) and project scale (0.31)
Team experience	0.51	0.035	14.57	< 0.001	Budget investment (0.68) and project scale (0.31)
Project scale	0.31	0.026	12.03	< 0.001	Budget investment (0.68) and team experience (0.51)
Budget investment × team experience	0.19	0.018	10.65	< 0.001	–
Budget investment × project scale	0.12	0.013	9.21	0.002	–
Team experience × project scale	0.08	0.009	8.94	0.003	–
Budget investment × team experience × project scale	0.06	0.007	8.17	0.005	–

In Table 4, the regression analysis results reveal that budget investment and team experience exert a significantly positive impact on RA outcomes. This suggests that increasing budget allocation and assembling a team with extensive experience can enhance project RA outcomes. Specifically, the regression coefficient for budget investment is 0.68, and for team experience, it is 0.51, both demonstrating significant positive effects (P < 0.05). The P-values are all significantly less than 0.05, indicating a significant impact. The impact of project scale is relatively small, at 0.31, and its P-value is also much less than 0.05. The degree of interaction influence is as follows. The impact of interaction terms is also significant, especially the interaction between budget investment and team experience and the interaction between budget investment and project scale. The P value of the interaction between budget investment and project scale is 0.002, and the P value of the interaction between team experience and project scale is 0.003. The P value of the interaction among budget investment, team experience, and project scale is 0.005. So, there are complex relationships and interactions among different factors, and budget investment and team experience significantly affect the RA results. However, the budget investment and project scale slightly affect the RA results. Project managers should comprehensively consider the interactive effects of different factors when making decisions to more accurately assess the risks of SRPs.

### The interaction between team experience and budget investment

The results of the interaction between team experience and budget investment are demonstrated in Table 5.

**Table 5:** Regression analysis of the degree of influence and interaction of different factors.

Team experience	Budget investment	Average RA results	Standard deviation	P Value (P < 0.05)
High	High	0.895	0.012	< 0.001
High	Medium	0.891	0.013	< 0.001
High	Low	0.885	0.015	< 0.001
Medium	High	0.898	0.011	< 0.001
Medium	Medium	0.894	0.012	< 0.001
Medium	Low	0.892	0.014	< 0.001
Low	High	0.905	0.013	< 0.001
Low	Medium	0.902	0.014	< 0.001
Low	Low	0.897	0.015	< 0.001

From Table 5, the degree of interaction impact can be obtained. Budget investment and team experience, along with the interaction between project scale and technical difficulty, are critical factors in risk mitigation. Particularly in scenarios characterized by large project scales and high technical difficulties, adequate budget allocation and a skilled team can substantially reduce project risks. As depicted in Table 5, under conditions of high team experience and sufficient budget investment, the average RA outcome is 0.895 with a standard deviation of 0.012, significantly lower than assessment outcomes under other conditions. This highlights the synergistic effects of budget investment and team experience in effectively mitigating risks in complex project scenarios. The interaction between team experience and budget investment has a significant impact on RA results. Under high team experience, the impact of different budget investment levels on RA results is not significant, but under medium and low team experience, the impact of different budget investment levels on RA results is significantly different. The joint impact of team experience and budget investment is as follows. Under high team experience, the impact of budget investment is relatively small, possibly because high-level team experience can compensate for the risks brought by insufficient budget to some extent. Under medium and low team experience, the impact of budget investment is more significant, possibly because the lack of team experience makes budget investment play a more important role in RA. Therefore, team experience and budget investment interact in RA of SRPs. They need to be comprehensively considered in project decision-making. High team experience can compensate for the risks brought by insufficient budget to some extent, but in the case of low team experience, the impact of budget investment on RA is more significant. An exhaustive consideration of these factors and their interplay is imperative for effectively assessing the risks inherent in SRPs. Merely focusing on budget allocation or team expertise may not yield a thorough risk evaluation. Project managers must scrutinize the project’s scale, technical complexity, and team proficiency, integrating these aspects with budget allocation and team experience. This holistic approach fosters a more precise RA and facilitates the development of tailored risk management strategies, thereby augmenting the project’s likelihood of success. In conclusion, acknowledging the synergy between budget allocation and team expertise, in conjunction with other pertinent factors, is pivotal in the RA of SRPs. Project managers should adopt a comprehensive outlook to ensure sound decision-making and successful project execution.

### Risk mitigation strategies

To enhance the discourse on project risk management in this paper, a dedicated section on risk mitigation strategies has been included. Leveraging the insights gleaned from the predictive model regarding identified risk factors and their corresponding risk levels, targeted risk mitigation measures are proposed.

Primarily, given the significant influence of budget investment and team experience on project RA outcomes, project managers are advised to prioritize these factors and devise pertinent risk management strategies.

For risks stemming from budget constraints, the adoption of flexible budget allocation strategies is advocated. This may involve optimizing project expenditures, establishing financial reserves, or seeking additional funding avenues.

In addressing risks attributed to inadequate team experience, measures such as enhanced training initiatives, engagement of seasoned project advisors, or collaboration with experienced teams can be employed to mitigate the shortfall in expertise.

Furthermore, recognizing the impact of project scale, duration, and technical complexity on RA outcomes, project managers are advised to holistically consider these factors during project planning. This entails adjusting project scale as necessary, establishing realistic project timelines, and conducting thorough assessments of technical challenges prior to project commencement.

These risk mitigation strategies aim to equip project managers with a comprehensive toolkit for effectively identifying, assessing, and mitigating risks inherent in SRPs.

## Discussion

This paper delves into the efficacy of the TANB algorithm in project risk prediction. The findings indicate that the algorithm demonstrates commendable performance across diverse projects, boasting high precision, recall rates, and AUC values, thereby outperforming analogous algorithms. This aligns with the perspectives espoused by Asadullah et al.^37^. Particular emphasis was placed on assessing the impact of variables such as budget investment levels, team experience, and project size on algorithmic performance. Notably, heightened budget investment and extensive team experience positively influenced the results, with project size exerting a comparatively minor impact. Regression analysis elucidates the magnitude and interplay of these factors, underscoring the predominant influence of budget investment and team experience on RA outcomes, whereas project size assumes a relatively marginal role. This underscores the imperative for decision-makers in projects to meticulously consider the interrelationships between these factors for a more precise assessment of project risks, echoing the sentiments expressed by Testorelli et al.^38^.

In sum, this paper furnishes a holistic comprehension of the Naive Bayes algorithm’s application in project risk prediction, offering robust guidance for practical project management. The paper’s tangible applications are chiefly concentrated in the realm of RA and management for SRPs. Such insights empower managers in SRPs to navigate risks with scientific acumen, thereby enhancing project success rates and performance. The paper advocates several strategic measures for SRPs management: prioritizing resource adjustments and team training to elevate the professional skill set of team members in coping with the impact of team experience on risks; implementing project scale management strategies to mitigate potential risks by detailed project stage division and stringent project planning; addressing technical difficulty as a pivotal risk factor through assessment and solution development strategies; incorporating project cycle adjustment and flexibility management to accommodate fluctuations and mitigate associated risks; and ensuring the integration of data quality management strategies to bolster data reliability and enhance model accuracy. These targeted risk responses aim to improve the likelihood of project success and ensure the seamless realization of project objectives.

## Conclusion

### Achievements

In this paper, the application of Naive Bayesian algorithm in RA of SRPs is deeply explored, and the influence of various factors on RA results and their relationship is comprehensively investigated. The research results fully prove the good accuracy and applicability of Naive Bayesian algorithm in RA of science and technology projects. Through probability estimation, the risk level of the project can be estimated more accurately, which provides a new decision support tool for the project manager. It is found that budget input and team experience are the most significant factors affecting the RA results, and their regression coefficients are 0.68 and 0.51 respectively. However, the influence of project scale on the RA results is relatively small, and its regression coefficient is 0.31. Especially in the case of low team experience, the budget input has a more significant impact on the RA results. However, it should also be admitted that there are some limitations in the paper. First, the case data used is limited and the sample size is relatively small, which may affect the generalization ability of the research results. Second, the factors concerned may not be comprehensive, and other factors that may affect RA, such as market changes and policies and regulations, are not considered.

The paper makes several key contributions. Firstly, it applies the Naive Bayes algorithm to assess the risks associated with SRPs, proposing the TANB and validating its effectiveness empirically. The introduction of the TANB model broadens the application scope of the Naive Bayes algorithm in scientific research risk management, offering novel methodologies for project RA. Secondly, the study delves into the impact of various factors on RA for SRPs through MLR analysis, highlighting the significance of budget investment and team experience. The results underscore the positive influence of budget investment and team experience on RA outcomes, offering valuable insights for project decision-making. Additionally, the paper examines the interaction between team experience and budget investment, revealing a nuanced relationship between the two in RA. This finding underscores the importance of comprehensively considering factors such as team experience and budget investment in project decision-making to achieve more accurate RA. In summary, the paper provides crucial theoretical foundations and empirical analyses for SRPs risk management by investigating RA and its influencing factors in depth. The research findings offer valuable guidance for project decision-making and risk management, bolstering efforts to enhance the success rate and efficiency of SRPs.

This paper distinguishes itself from existing research by conducting an in-depth analysis of the intricate interactions among various factors, offering more nuanced and specific RA outcomes. The primary objective extends beyond problem exploration, aiming to broaden the scope of scientific evaluation and research practice through the application of statistical language. This research goal endows the paper with considerable significance in the realm of science and technology project management. In comparison to traditional methods, this paper scrutinizes project risk with greater granularity, furnishing project managers with more actionable suggestions. The empirical analysis validates the effectiveness of the proposed method, introducing a fresh perspective for decision-making in science and technology projects. Future research endeavors will involve expanding the sample size and accumulating a more extensive dataset of SRPs to enhance the stability and generalizability of results. Furthermore, additional factors such as market demand and technological changes will be incorporated to comprehensively analyze elements influencing the risks of SRPs. Through these endeavors, the aim is to provide more precise and comprehensive decision support to the field of science and technology project management, propelling both research and practice in this domain to new heights.

### Limitations and prospects

This paper, while employing advanced methodologies like TANB models, acknowledges inherent limitations that warrant consideration. Firstly, like any model, TANB has its constraints, and predictions in specific scenarios may be subject to these limitations. Subsequent research endeavors should explore alternative advanced machine learning and statistical models to enhance the precision and applicability of RA. Secondly, the focus of this paper predominantly centers on the RA for SRPs. Given the unique characteristics and risk factors prevalent in projects across diverse fields and industries, the generalizability of the paper results may be limited. Future research can broaden the scope of applicability by validating the model across various fields and industries. The robustness and generalizability of the model can be further ascertained through the incorporation of extensive real project data in subsequent research. Furthermore, future studies can delve into additional data preprocessing and feature engineering methods to optimize model performance. In practical applications, the integration of research outcomes into SRPs management systems could provide more intuitive and practical support for project decision-making. These avenues represent valuable directions for refining and expanding the contributions of this research in subsequent studies.

### Supplementary Information

Supplementary Information.

## Data Availability

All data generated or analysed during this study are included in this published article [and its Supplementary Information files].

## References

[1] Moshtaghian F, Golabchi M, Noorzai E (2020). A framework to dynamic identification of project risks. Smart and sustain. Built. Environ..

[2] Nunes M, Abreu A (2020). Managing open innovation project risks based on a social network analysis perspective. Sustainability.

[3] Elkhatib M, Al Hosani A, Al Hosani I (2022). Agile project management and project risks improvements: Pros and cons. Mod. Econ..

[4] Fridgeirsson TV, Ingason HT, Jonasson HI (2021). The VUCAlity of projects: A new approach to assess a project risk in a complex world. Sustainability.

[5] Salahuddin T (2023). Numerical Techniques in MATLAB: Fundamental to Advanced Concepts.

[6] Awais M, Salahuddin T (2024). Radiative magnetohydrodynamic cross fluid thermophysical model passing on parabola surface with activation energy. Ain Shams Eng. J..

[7] Awais M, Salahuddin T (2023). Natural convection with variable fluid properties of couple stress fluid with Cattaneo-Christov model and enthalpy process. Heliyon.

[8] Guan L, Abbasi A, Ryan MJ (2020). Analyzing green building project risk interdependencies using Interpretive Structural Modeling. J. Clean. Prod..

[9] Gaudenzi B, Qazi A (2021). Assessing project risks from a supply chain quality management (SCQM) perspective. Int. J. Qual. Reliab. Manag..

[10] Lee KT, Park SJ, Kim JH (2023). Comparative analysis of managers’ perception in overseas construction project risks and cost overrun in actual cases: A perspective of the Republic of Korea. J. Asian Archit. Build. Eng..

[11] Garai-Fodor M, Szemere TP, Csiszárik-Kocsir Á (2022). Investor segments by perceived project risk and their characteristics based on primary research results. Risks.

[12] Senova A, Tobisova A, Rozenberg R (2023). New approaches to project risk assessment utilizing the Monte Carlo method. Sustainability.

[13] Tiwari P, Suresha B (2021). Moderating role of project innovativeness on project flexibility, project risk, project performance, and business success in financial services. Glob. J. Flex. Syst. Manag..

[14] de Araújo F, Lima P, Marcelino-Sadaba S, Verbano C (2021). Successful implementation of project risk management in small and medium enterprises: A cross-case analysis. Int. J. Manag. Proj. Bus..

[15] Obondi K (2022). The utilization of project risk monitoring and control practices and their relationship with project success in construction projects. J. Proj. Manag..

[16] Atasoy G, Ertaymaz U, Dikmen I (2022). Empowering risk communication: Use of visualizations to describe project risks. J. Constr. Eng. Manage..

[17] Dandage RV, Rane SB, Mantha SS (2021). Modelling human resource dimension of international project risk management. J. Global Oper. Strateg. Sourcing.

[18] Wang L, Sun T, Qian C (2020). Applying social network analysis to genetic algorithm in optimizing project risk response decisions. Inf. Sci..

[19] Marx-Stoelting, P. *et al.* A walk in the PARC: developing and implementing 21st century chemical risk assessment in Europe. *Arch. Toxicol.* **97**(3), 893–908 (2023). 10.1007/s00204-022-03435-7 PMC9968685 36645448

[20] Awais M, Salahuddin T, Muhammad S (2023). Evaluating the thermo-physical characteristics of non-Newtonian Casson fluid with enthalpy change. Thermal Sci. Eng. Prog..

[21] Awais M, Salahuddin T, Muhammad S (2024). Effects of viscous dissipation and activation energy for the MHD Eyring-Powell fluid flow with Darcy-Forchheimer and variable fluid properties. Ain Shams Eng. J..

[22] Yang L, Lou J, Zhao X (2021). Risk response of complex projects: Risk association network method. J. Manage. Eng..

[23] Acebes F, Pajares J, González-Varona JM (2021). Project risk management from the bottom-up: Activity Risk Index. Cent. Eur. J. Oper. Res..

[24] Siyal S, Saeed M, Pahi MH (2021). They can’t treat you well under abusive supervision: Investigating the impact of job satisfaction and extrinsic motivation on healthcare employees. Rationality Society.

[25] Chen D, Wawrzynski P, Lv Z (2021). Cyber security in smart cities: A review of deep learning-based applications and case studies. Sustain. Cities Soc..

[26] Zhao M, Wei G, Wei C (2021). Pythagorean fuzzy TODIM method based on the cumulative prospect theory for MAGDM and its application on risk assessment of science and technology projects. Int. J. Fuzzy Syst..

[27] Suresh K, Dillibabu R (2020). A novel fuzzy mechanism for risk assessment in software projects. Soft Comput..

[28] Akhavan M, Sebt MV, Ameli M (2021). Risk assessment modeling for knowledge based and startup projects based on feasibility studies: A Bayesian network approach. Knowl.-Based Syst..

[29] Guan L, Abbasi A, Ryan MJ (2021). A simulation-based risk interdependency network model for project risk assessment. Decis. Support Syst..

[30] Vujović V, Denić N, Stevanović V (2020). Project planning and risk management as a success factor for IT projects in agricultural schools in Serbia. Technol. Soc..

[31] Muñoz-La Rivera F, Mora-Serrano J, Oñate E (2021). Factors influencing safety on construction projects (FSCPs): Types and categories. Int. J. Environ. Res. Public Health.

[32] Nguyen PT, Nguyen PC (2020). Risk management in engineering and construction: A case study in design-build projects in Vietnam. Eng. Technol. Appl. Sci. Res.

[33] Nguyen PT, Le TT. Risks on quality of civil engineering projects-an additive probability formula approach//AIP Conference Proceedings. AIP Publishing, 2798(1) (2023).

[34] Nguyen, P.T., Phu, P.C., Thanh, P.P., *et al*. Exploring critical risk factors of office building projects. **8**(2), 0309–0315 (2020).

[35] Nguyen HD, Macchion L (2023). Risk management in green building: A review of the current state of research and future directions. Environ. Develop. Sustain..

[36] He S, Xu H, Zhang J (2023). Risk assessment of oil and gas pipelines hot work based on AHP-FCE. Petroleum.

[37] Asadullah M, Hossain MM, Rahaman S, Amin MS, Sumy MSA, Parh MYA, Hossain MA (2023). Evaluation of machine learning techniques for hypertension risk prediction based on medical data in Bangladesh. Indones. J. Electr. Eng. Comput. Sci..

[38] Testorelli R, de Araujo F, Lima P, Verbano C (2022). Fostering project risk management in SMEs: An emergent framework from a literature review. Prod. Plan. Control.

